# PRE-CLINICAL SKILLS: A competency-based assessment integrated course implemented early in the curriculum to prepare second-year medical students prior to entering clinical settings

**DOI:** 10.15694/mep.2017.000110

**Published:** 2017-06-21

**Authors:** Maria Rosa Fenoll-Brunet, Verónica Piera, Domènec Sánchez, Maria Angel Lanuza, Neus García, Pedro Cobos

**Affiliations:** 1 University Rovira i Virgili

**Keywords:** Undergraduate Medical Education, Pre-clinical skills, Competence-based assessment, Basic science education, Reference standards

## Abstract

This article was migrated. The article was marked as recommended.

**Objective:** This *Case Study* describes the experience of *Rovira i Virgili* University School of Medicine (URV) with the early introduction of pre-clinical skills learning in the undergraduate medical curricula to monitor and assessing these competencies as a prerequisite for medical students accessing their training in clinical settings.

**Course Development:** A PRE-CLINICAL SKILLS course has been developed to guarantee medical student’s performance in managing clinically relevant basic medical sciences to interpret clinical scenarios, to develop technical communication skills and to value professional behavior throughout the first two years of medical education. The set of pre-clinical competencies evaluated in this course as well as the corresponding assessment methods have been established according to an international reference standards review work in collaboration with the regional quality assurance agency. An integrated formative assessment is being used.

**Course Advantages, Added Values and Outcome Measures:** Since the academic year 2009-2010 about 130 students from second-year of Medicine follows this integrated trunk-course while being enrolled in parallel in other core courses as Physiology, Anatomy, and Histology. The program doesn’t include lectures but only learning activities to train and monitor the successful achievement of the pre-clinical skills by medical students. A good majority of the participants achieve readiness for start training in clinical settings. As a whole, this course is useful ensuring patient’s safety by identifying weaknesses acquisition of pre-clinical skills and predicting medical students who will have difficulty during their clinical training.

**Conclusions:** Reflecting on our experience, we believe that the named course “PRE-CLINICAL SKILLS” overcomes the disadvantages of the traditional teaching methods. Helping students to conceptualize rather than memorize and encouraging them to integrate clinically relevant basic medical sciences concepts and principles by training pre-clinical skills in this competence-based assessment course prior entering into clinical settings.

## Introduction

In most pre-clinical courses a considerable amount of laboratory practices are carried out, however, the degree of acquisition of related skills is not usually subject to evaluation, or they have little impact on the final score. Moreover, the assessment of the clinical competencies is traditionally still been assessed mainly on the final courses.

With these issues in mind, the course “PRE-CLINICAL SKILLS” has been designed to overcome the disadvantages of the traditional teaching and assessment methods into a traditional lecture-based accredited curriculum of Medicine. The main objective of the course “PRE-CLINICAL SKILLS” is to evaluate essential pre-clinical skills as a prerequisite to access training in health centers. Thus, ensuring that medical students can adequately deal with the next stage of their training. Looking for supporting learners integrating the study of basic life sciences including anatomy, physiology, histology but also genetics, ethics, professionalism or biochemistry; promoting teaching activities to conceptualize rather than memorize clinically relevant knowledge and, encouraging the integration of the clinically relevant basic sciences concepts and principles into the clinical sciences. Through inter-disciplinary learning activities and a competence-based integrative assessment.

## Course Development

The PRE-CLINICAL SKILLS course has been developed to assess performance in managing basic sciences knowledge to interpret clinical scenarios, communication skills and professional behavior throughout the first two undergraduate years.

The set of pre-clinical competencies evaluated in this course ( AQU Catalunya, 2004) and their corresponding competence-assessment methods ( AQU Catalunya, 2009) have been established according to frameworks developed by the Catalan Medical Schools of Medicine with a team of experienced medical education experts in assessment at undergraduate and postgraduate level under the *DISSENY* project granted by the Catalan University Quality Assurance Agency, AQU *Catalunya.* AQU is a full member of the European Association for Quality Assurance in Higher Education (ENQA) and AQU is also a member of the International Network for Quality Assurance Agencies in Higher Education (INQAAHE).

### “PRE-CLINICAL SKILLS” course competence and assessment frameworks:

The framework of essential competencies/learning outcomes for undergraduate medical Education ( AQU Catalunya, 2004) is grounded on the “Global Minimum Essential Requirements in Medical Education” issued by the Institute for International Medical Education ( IIME Core Committee, 2002). Whereas more than 300 articles and reference documents were reviewed to elaborated the “Guide for Evaluation of Medical Competences” ( AQU Catalunya, 2009) which includes: 1. the analysis of the main medical catalogues of competencies/learning outcomes, 2. the process of assessment of medical competencies (international, national and local context, strategies and procedures), and 3. Recommendations for the assessment of those competencies/outcomes previously established. These outcomes were presented at AMEE 2009 by the Professor Jose Carreras as coordinator of these AQU projects.

### Training method:

Active learning is promoted and skills training is geared to the curriculum to ensure that the training sessions deal with subjects that are addressed in simultaneous basic medical sciences modules. Students are assigned to training sessions in small groups. Moreover, regular tutorials are offered with no limited duration.

This “PRE-CLINICAL SKILLS” course has been launched in 2010 and is based on the incorporation of simple and tested educational strategies such as case-based instruction, team-based learning (TBL) and interactive learning. As strategies to encourage active learning we used interactive, flipped and blended approaches. Students are trained in small groups to elaborate written reports in histology laboratories, to discuss case studies and radiological anatomy images in TBL format, a variety of competencies are trained in the skills lab. For instance, the radiological anatomy seminars are interactive and promote discussion of cases; the concept map is used as a tool for educational strategy applied to basic health sciences, particularly in the area of histology and as a self-learning resource as well as quizzes allowing learners to learn in an entertaining environment and to extend their learning beyond the teaching rooms. Students trained in small groups discuss case studies, learn to elaborate written reports on histology images, learn radiological anatomy on medical images, and train pre-clinical skills different training labs, microscopy rooms and virtual microscopy laboratories. Where students start gaining the ability to transfer basic medical knowledge, to reflect on clinical reports and to interpret laboratory and clinical test results in a way the students realize that they can use basic sciences to understand clinical scenarios.

The educational portal “Moodle” is used as open-source learning platform and course management tool. Providing educational resources, including brain maps, web pages, practice guidelines, discussion forums, and educational videos. Also, hosts a short video presentation of this course that is also available on YouTube ( https://www.youtube.com/watch?v=0zF_VnnNyZA). Resulting from a collaborative effort between all teaching staff. Learning objectives are clearly identified before each teaching session. Preparatory pieces of advice are suggested by the faculty and are posted on Moodle. The pieces of advice comprise information about objectives, contents, and methods of each training session.

The course is being developed teachers of the core basic sciences (Anatomy, Physiology, and Histology) and teachers of the clinical sciences (Surgery, Radiology), those who design and evaluate.

### Integrated competence-based formative assessment:

We have identified a set of core pre-clinical competencies that all participants have to reach 100%. We chose not to include all of them in this manuscript however they are available for enrolled trainees at Moodle. See the “PRE-CLINICAL SKILLS” course overview in Table 1.

A small-group teaching pre-clinical competencies course in a non-integrated curriculum to monitor and assess these skills as a prerequisite for these medical students accessing their training in clinical settings. Thus, the set of pre-clinical skills achievement is compulsory.

An integrated formative assessment is being used and includes two parts:

First, a clinical scenario on skills-related theory serves as the basis for developing 30 multiple choice questions. Discrete items that align to the areas of competence described in the Catalan Curriculum Framework (five are associated with the interpretation of medical images).

Second, a tour through five skills-assessment stations allows to individually evaluate the acquisition of the set of essential pre-clinical skills and level attained according to rubrics.

The assessment for each student ran for 45 minutes to answer the “short-case” by a multiple-Choice 30-items questionnaire. During that time all students were presented with one ‘short case’ and that were unique for each student cohort. The diagnostic hypotheses guide this reflective exam. Thereafter, students follow a route and stops for 20 minutes in five assessment stations. We have identified core competencies that all participants have to reach 100%. The systematic exam includes them all:

1. Performing cardiopulmonary resuscitation (RCP) and Heimlich maneuvers
2. Physical exam: cranial nerves examination, heart and lung auscultation, reflexes, arterial pulses and abdominal exploration
3. Differentiating between physiological and pathological medical images: ECG, X-Rays, TC, RMN
4. Differentiating between physiological and pathological blood cells, basic tissues from histological images, spirometry results, blood tests results
5. Writing a technical report of a healthy human histology specimen describing cells and tissue organization

The course “PRE-CLINICAL SKILLS” evaluates essential clinical skills as a prerequisite to ensure that students can adequately deal with the next stage of their training, work placements in institutions. By using the recommendations on assessment of competencies/learning outcomes in Medicine that have published academic and professional state and international organizations, as well as the recommendations of medical education associations in Spain and Catalonia (2009). Furthermore, we created our own set of case-based inter-disciplinary formative assessment. Where the second-year medical student is never asked for a diagnosis or management about a chief complaint, but the history of present illness and medical history are explained, physical examination, and diagnostic workup are presented in a succinct way leading to a question for the learner about to implement clinically relevant basic medical sciences. Excellent clinical teacher’s participation ensures readability, style, and content before the assessment material is being used. The goal has been to develop a storehouse of clinical scenarios to motivate medical students to learn more about clinically relevant life sciences, to ensure their readiness for clinical years and also practice acquired in handling glass slides and a microscope and writing reports. But also to start assessing reasoning abilities on ethical dilemmas in a medium-low-cognitive level.

**Figure 1.**
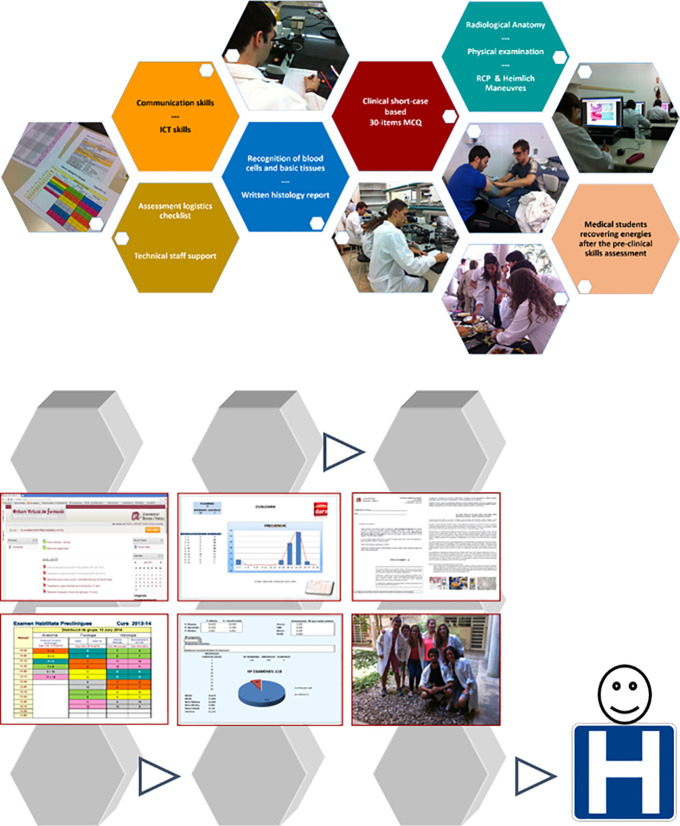
The “PRE-CLINICAL SKILLS” course logistics

Short case scenario and grading rubric are developed to assess respondents’ behavior and basic sciences knowledge application in Exploring patient clinical tests, and interaction with patient’s diagnostic images ( Figure 1 shows logistics and few examples). At least, a 10-item scale is developed to assess how respondents explored patients’ clinical contexts. Response options were determined, following clinical teachers. Scores for items ranged from 0-1. After the problem-based scenarios that present aspects of a case in steps, each one requiring recording of student reasoning and investigation of knowledge sources for solutions to queries, the students follow a set of five assessment stations where they have to perform written, verbal or practical performances.

### Examiners:

All examiners are clinical and non-clinical teachers with over 10 years’ experience in assessments and assessment design. Ensuring also that teachers with the best clinical skills are being recruited. Moreover, we foster positive lifelong professional relationships with excellent clinical teachers.

### Course participants:

The study population consisted of medical undergraduate students in the pre-clinical phase of their education. All medical students in the two years of their six-year accredited program participated as “Pre-Clinical Skills” is this is a core course. Pre-Clinical Skills training session’s group size varied from six to twelve students. Whereas radiological anatomy seminars for case-based discussion are larger. According to personal learning style, some students preferred studying assigned preparatory work before or after the training sessions. All entry students from other medical curricula have to compulsory pass this course before entering the clinical courses.

## Course Advantages, Added Values and Outcome Measures

Since the academic year 2009-2010, around 130 undergraduate medical students of the second year of Medicine enrolled this “PRE-CLINICAL SKILLS” course at the University *Rovira i Virgili* (Catalonia, Spain). While being enrolled in parallel in traditional core courses on Physiology, Anatomy, and Histology. This school offers a six-year undergraduate-entry curriculum.

The course is being scheduled towards the end of the second semester. Students are distributed in small groups and one tutor is assigned per group. This academic activity is carried out by professors of Anatomy, Physiology and Histology of the Department of Basic Medical Sciences, some of which are linked to university teaching hospitals (i.e., experienced bedside teachers from the following services: Surgery, Radiology, Intensive Care and/or Traumatology).

The training program doesn’t include lectures only active learning activities to train and monitor the successful achievement of the pre-clinical skills by medical students. As a whole, this course offers many learning resources and serves to facilitate the acquisition of the pre-clinical skills before their assessment. Trainees are individually trained in the physiology skills labs, in small groups in seminars for case discussions to correlate anatomy to clinical radiological images and in both, optical microscopy room’s and virtual histology labs to train the identification of blood cells and basic tissues as well as for training the preparation of well-structured written reports. Table 1 summarizes the “PRE-CLINICAL SKILLS” course overview, including the different learning spaces used.

This course identifies the essential pre-clinical competencies to be evaluated as well as the qualification criteria. Taking into account the regional medical education competence framework established by the Quality Assurance Agency in Catalonia (2009).

### Advantages and benefits:

• Despite the challenge of assessing all the practical skills of the first and second years of Medicine in this course, the students leave a little stressed but very satisfied with the experience.
• Formative assessment methods are used. In addition, where students can demonstrate the usefulness of applying their knowledge to the basic medical sciences into clinical scenarios.
• Guidance for the development of a pre-clinical competency training program: Assessing pre-clinical competencies of medical student’s prior entering clinical setting allows for the identification of gaps in knowledge and appropriate behavior that reflect specific areas for improvement of their medical Education before accessing training in clinical settings.

### Concerns related to course implementation:

As added value, we found that the development integrative assessment on basic medical sciences resulted very useful to identify existing gaps in competence training in the curricula. Thus, enabling improvements when necessary. Horizontal and vertical content integration has to be promoted in medical education and introduced in teaching-learning methods, rather than assuming that students somehow integrate knowledge on their own ( Dent and Harden, 2005).

### Outcome measures:

We feel particularly proud of the undergraduate medical students enrolled in this course for their achievements in their clerkships ( Castro et al. 2015). Very especially of Mr. Ramón Bultó, a trainee of this course who won the RCP competition at AMEE Milan in 2014.

## Discussion

A competence-based course for early train pre-clinical medical students can ensure that undergraduate medical students can appropriately deal with the next stage of their training, work placements in health care institutions. Furthermore, ensuring that physical examination skills are no longer declining among medical trainees, as many institutions are not teaching these systematically and effectively ( Ramani, 2008).

We shortly described our experience in designing an integrated basic medical sciences academic activity for teaching-learning and evaluating the acquisition of pre-clinical skills by undergraduate medical students of the second year into a non-integrated curriculum of Medicine in order to overcome the disadvantages of the traditional teaching methods. The named course “PRE-CLINICAL SKILLS” was designed approaching clinically relevant basic medical sciences to clinical practice. The main objectives of the course are helping students to acquire pre-clinical skills, to conceptualize rather than memorize knowledge and encouraging participants to integrate basic medical sciences into clinical scenarios looking for their optimal preparation for the clinical training. Thus, ensuring the readiness of pre-clinical medical students to successfully develop clinical skills in clinical settings. Implementation of a pre-clinical fundamental skills curriculum appears to be associated with improved clerkship performance in the 3rd year of medical school, particularly in the Internal Medicine clerkship ( Jackson et al., 2009).

The pre-clinical competencies we evaluate and their corresponding assessment methods emerge from two previous review works promoted by the regional quality assurance agency (AQU-Catalunya). The assessment methods used to allow to evaluate not only the achievement of pre-clinical skills but also the use of basic medical sciences knowledge for the understanding of the basic and clinical concepts, the capacity for clinical reasoning and the ability to apply basic knowledge to clinical problems. Each academic year a clinical scenario is created, the same for all students, so that they are all evaluated by asking the same questions and using the same checklists and the same criteria, standards, and scales previously set and taking into account foreseeable responses.

Continued efforts are needed to understand how to best prepare students for clinical clerkships and how to evaluate outcomes of similar pre-clinical skills programs ( Jackson et al., 2009). Also considered what drives medical students to prepare or not to prepare clinical skills ( Aalbers et al. 2013). We yearly tried to introduce course improvements, starting from taking into account the twelve tips for excellent physical examination teaching described by Subha Ramani (2008). Our aim as trainers is to continue improving this course and by next academic year, we’ll also like to ensure following the recently published medical education articles on Medical Teachers. These are Twelve tips on writing a discussion case that facilitates teaching and engages learners ( Cohen et al, 2017) and Twelve tips for the construction of ethical dilemma case-based assessments ( Tsai, 2017). As discussion sessions based on clinical cases are well known to promote active learning, critical thinking, and higher order processing. However, if the case is poorly written, then the session can be difficult to lead and met with silence rather than rich learner engagement.

## Conclusion

Reflecting on our experience, we believe that the named course “PRE-CLINICAL SKILLS” overcomes the disadvantages of the traditional teaching methods. Helping students to conceptualize rather than memorize and encouraging them to integrate clinically relevant basic medical sciences concepts and principles by training pre-clinical skills in this competence-based assessment course prior entering into clinical settings.

## Take Home Messages

• Integrating life-sciences into a non-integrated curriculum is possible even in a traditional lecture-based curriculum of Medicine.
• Pre-clinical skills must be strongly recommended to be acquired before entering clinical courses in the undergraduate Medical curriculum. Having patient safety in mind, medical students should demonstrate their competency on essential clinical skills in the first level of competence before entering clinical settings.
• A “PRE-CLINICAL SKILLS” course including active learning activities and formative assessment can ensure the acquisition of these skills and motivates participants to learn the usefulness of applying basic medical sciences knowledge in clinical scenarios. The early achievement of pre-clinical skills might be even used earlier allowing pre-clinical students to respond adequately to a medical emergency that might happen in their everyday life.
• Finally, assessing pre-clinical competencies of medical students that have completed the second year of the six-year curriculum can provide indicators on the early effectiveness of competence training in that curriculum and to early identify inappropriate behaviors. Such data can be used to guide further efforts revising and improving the curricula.

## Notes On Contributors

**Maria Rosa Fenoll-Brunet** MD Ph.D. is a full Professor of Histology at URV School of Medicine.

**Domènec Sánchez** BD, Ph.D. is a full Professor of Physiology at URV School of Medicine.

**Verònica Piera** MD, Ph.D. MD is a full Professor of Anatomy at URV School of Medicine.

**María Angel Lanuza** BD, Ph.D. is a full Professor of Histology at URV School of Medicine.

**Neus García** BD, Ph.D. is a full Professor of Histology at URV School of Medicine.

**Pedro Cobos** MD, Ph.D. is a Senior Professor of Anatomy at URV School of Medicine, Surgeon and former Chair of the Surgery service at the University Teaching Hospital John XXIII.

## Appendices

**Table 1.** The “PRE-CLINICAL SKILLS” course overview

Training activities			
What do we train: *Clinically relevant basic medical sciences according to the AQU guide of professional competences*	*How do we train* *: Exclusively through student’s active learning and hands-on training activities. No lectures are held.*		
	Knowledge	Skills	Learning space
**Integrating:** • Anatomy • Physiology • Histology	Anatomy and normal radiological images (X-Ray, TC, RMN)	Skills to identify normal vs pathological macroscopically images	Anatomy skills lab
	First-aid main maneuvers Full physical examination Electrocardiogram and spirometry principles and management Laboratory analysis tests	Skills for RCP & Heimlich maneuvers Skills performing a physical exam, to proper using an electrocardiogram (ECG) and a spirometry equipment Skills identifying normal vs pathological results of ECGs, spirometries Skills interpreting laboratory data	Physiology skills lab
	Optical microscope management Recognition of healthy blood cells, basic histological tissues, and organs. Main histological stains	Skills for handling an optical microscope Skills identifying normal vs pathological cells and tissue microscopically images Skills preparing a technical report describing cells and tissues	Histology skills lab: Microscopes and virtual microscopy
**Other fields integrated**	Biochemistry, Genetics, Ethics, Biology, Professionalism	Skills for data gathering	Team-Based Learning (TBL) seminar
**Competence-based assessment**			
***How we measure*** *: according to the AQU guide of methodologies for* competence assessment *and aligning assessment to course learning objectives ( AQU Catalunya, 2009)*			
**Type of assessment**			
30-items Multiple Choice Questionnaire	*Answers on a 2-point scale:* • Case-based questions with ‘correct’ and ‘incorrect’ response options (10 items) *Answers on a 5-point scale:* • 10-item scale on knowledge of patients’ individual clinical context • Exploring professionalism behavior preference in critical clinical scenarios (2 items) • *Culturally competent consultation behavior (1 item)* Five items are associated with the interpretation of medical images		
Skills lab assessment	*All five skills-assessment stations have their checklist scores and pass rates for each pre-clinical skills assessed*		
**Performance evaluation domains**			
Attendance and participation	**Students have to perform 100% all parts**		
Procedural skills	Skills lab assessment Direct observation of procedural skills (DOPS)		
Technical communication skills	Objective structured practical examination (OSPE)		
Data gathering	Multiple Choice Questions (MCQ) Case-based discussion (CbD)		
Educational attitudes			
Communication skills	Practical structured (SOE)		

The collage of photos at the top shows a selection of the different assessment theme scenarios where students are being evaluated, including some of the mandatory pre-clinical skills assessed and a meaningful tool which is the “assessment logistics’ checklist”. At the bottom: a selection of images shows examples of different course activities, results and useful tools. Including Moodle, one-page “short-case” presentation and the two graphics showing the MCQ results, an example of a day-exam assessment by stations schedule and a team of very satisfied students once they have finished the assessment. The standing man medical student won the following year the RCP competition at AMEE in Milan (2014).
