# Supplementary material for: PRE-CLINICAL SKILLS: A competency-based assessment integrated course implemented early in the curriculum to prepare second-year medical students prior to entering clinical settings
Source: MedEdPublish (2016). 2017 Jun 21;6:110. [Version 1] doi: 10.15694/mep.2017.000110 (PMC10885266; doi:10.15694/mep.2017.000110)
Supplement: Peer review history

# Reviewer report: Reviewer response for version 1

**DOI:** 10.21956/mep.19270.r28009

**Published:** 2017-06-24

**Authors:** Barbara Jennings

**Affiliations:** 1 Norwich Medical School

This review has been migrated. The reviewer awarded 4 stars out of 5 I really enjoyed reading this article because the authors present an interesting and detailed reflection on the introduction and development of a pre-clinical skills strand to their 6 year medical degree programme. The authors explain how this has complemented both traditional teaching methods and the pre-clinical/clinical course structure. Active learning is encouraged through the use of case-based and team-based learning and the use of skills laboratories to explore sciences such as anatomy, histology and physiology. The authors explain that the use of this applied approach, even from the second semester onwards, means that “students realise that they can use basic sciences to understand clinical scenarios”. In their outcome measures, the authors cite a previous study that demonstrates the subsequent clerkship achievements of the students who completed this training. Table 1 in the appendix provides a really helpful overview of the aims and delivery of the formative assessments. There are some important aspects to the assessments used:• They are formative and assess defined core competences• They assess integrated skills across basic and clinical sciences• Students have to pass all (100 %) of tasksThese features appeal to me because the course team is taking care to develop a curriculum that is focused on the essential knowledge that underpins clinical skills; not taking up time with esoteric or provisional knowledge of life sciences that may not prove useful subsequently.A few suggestions for the manuscript: I think the syntax of the paper could have been improved in a few places and particularly in the abstract. Proof reading and careful consideration of English syntax/grammar before producing the final version may have helped to improve the clarity overall. The manuscript could also have been a bit more succinct; there was some repetition rather than development of ideas between sections. Two questions for the authors: Could you tell us more about how the formative assessment works – does it affect progression of the students? What proportion of students pass first time?I recommend this paper to life-sciences teachers, particularly if they are contributing to a medical degree with a traditional structure. When the authors submitted a preliminary draft of their manuscript they included a William Osler quotation that I liked very much, it reminded me that the notion of being realistic about a core curriculum and the importance of life-long learning are not new philosophies! I am a co-editor for this themed edition of the journal but have no other COIs.

Reviewer Expertise:

NA

No decision status is available

# Reviewer report: Reviewer response for version 1

**DOI:** 10.21956/mep.19270.r28008

**Published:** 2017-06-21

**Authors:** Trevor Gibbs

**Affiliations:** 1 AMEE

This review has been migrated. The reviewer awarded 3 stars out of 5 An interesting paper to read in 2017, which demonstrates the variability in curricula in different parts of the world. I think that anyone reading this paper from the UK, would be saying " we have been doing this for years' . The concept of the breaking down of the pre-clinical / clinical divide, early clinical exposure, an integrated, inter professional curriculum and an active approach to learning through active teaching and TEL were all begun from the first iteration of Tomorrows Doctors. However despite these reservations, I would congratulate the authors in trying to bring change to an outmoded method of curriculum structure. It would be better if the pre-clinical skills course was more integrated into all of the first two years, not as a stand alone course, and more appropriate methods of assessment were used rather than rely on MCQs

Reviewer Expertise:

NA

No decision status is available
